# Supplementary material for: Prioritization of surgical, obstetric, trauma, and anesthesia care in India over seven decades: A systematic analysis of policy documents
Source: PLOS Glob Public Health. 2023 Jul 31;3(7):e0002084. doi: 10.1371/journal.pgph.0002084 (PMC10389714; doi:10.1371/journal.pgph.0002084)
Supplement: S1 Table — (DOCX) [file pgph.0002084.s001.docx]

**Supplementary Information**

**Supplementary Table S1: List of documents included in the analysis.**

| **Sr No.** | **Document Name** | **Publication year** | **Description** | **Link** |
| --- | --- | --- | --- | --- |
| 1. | Bhore Report  [1–3] | 1946 | A comprehensive three volume report submitted by the Bhore Committee set up in 1943, by the Government of India, for appraisal of the condition of health services in the country. | <http://www.nihfw.org/Doc/Reports/bhore%20Committee%20Report%20VOL-1%20.pdf>  <http://www.nihfw.org/Doc/Reports/Bhore%20Committee%20Report%20-%20Vol%20II.pdf>  <http://www.nihfw.org/Doc/Reports/Bhore%20Committee%20Report-%203.pdf> |
| 2. | Mudaliar Report  [4] | 1962 | The 'Health Survey and Planning committee' headed by Dr. A. L. Mudaliar was formed to assess the progress made in the health sector post the recommendations made by the Bhore Committee. They submitted this report in 1962. | <https://www.nhp.gov.in/sites/default/files/pdf/Mudalier_Vol.pdf> |
| 3. | Mukerji 1 Report  [5] | 1965 | The Mukerji committee, headed by the then Union Health Secretary-Shri B. Mukherjee, provided recommendations in the areas of family planning and malaria operations through this report. | <https://www.nhp.gov.in/sites/default/files/pdf/Mukerjee_Committee_Report.pdf> |
| 4. | Mukerji 2 Report  [6] | 1966 | The 2nd part of the report of the same name, published in 1966. | <https://www.nhp.gov.in/sites/default/files/pdf/Mukerji_Committee_Report_PartII.pdf> |
| 5. | Jungalwalla Report  [7] | 1967 | This report was submitted in 1967 by the Jungalwalla Committee, also known as the 'Committee on integration of health services', headed by Dr. N Jungalwalla. | <https://www.nhp.gov.in/sites/default/files/pdf/Jungalwal_Committee_Report.pdf> |
| 6. | Kartar Singh Report  [8] | 1973 | The Committee on Multipurpose Workers under Health and Family Planning, headed by the Additional Secretary of Health-Kartar Singh, submitted this report in 1972. | <https://www.nhp.gov.in/sites/default/files/pdf/Kartar_Singh_Committee_Report.pdf> |
| 7. | Shrivastav Report  [9] | 1975 | The committee responsible for this report was set up in 1974 as 'Group on Medical Education and Support Manpower'. | <https://www.nhp.gov.in/sites/default/files/pdf/Srivastava_Committee_Report.pdf> |
| 8. | Bajaj Report  [10] | 1986 | The Ministry of Health and Family Welfare set up a committee on Health Manpower, Planning, Production and Management which compiled its recommendations in this report in 1986. | <https://www.nhp.gov.in/sites/default/files/pdf/Bajaj_Committee_report.pdf> |
| 9. | National Health Policy (NHP)- 1983  [11] | 1983 | First National Health Policy of India was formulated by the Ministry of Health and Family Welfare (MoHFW) to fulfill the goal of 'health for all' by the year 2000. | <https://www.nhp.gov.in/sites/default/files/pdf/nhp_1983.pdf> |
| 10. | National Health Policy (NHP)- 2002  [12] | 2002 | Second iteration of the National Health Policy. | <https://www.nhp.gov.in/sites/default/files/pdf/NationaL_Health_Pollicy.pdf> |
| 11. | National Health Policy (NHP)- 2017  [13] | 2017 | Latest version of the National Health Policy. | <https://www.nhp.gov.in/nhpfiles/national_health_policy_2017.pdf> |
| 12. | Planning Commission (PC) 1 [14] | 1951 | Health Section of the first Five Year Plan (1951-56), conceptualised by the Planning Commission of India. The Planning Commission was a body responsible for policy formulation and governance in the country. | <https://niti.gov.in/planningcommission.gov.in/docs/plans/planrel/fiveyr/1st/1planch32.html> |
| 13. | Planning Commission (PC) 2 [15] | 1956 | Health Section of the second Five Year Plan (1956-61) | <https://niti.gov.in/planningcommission.gov.in/docs/plans/planrel/fiveyr/2nd/2planch25.html> |
| 14. | Planning Commission (PC) 3 [16] | 1961 | Health Section of the third Five Year Plan (1961-66) | <https://niti.gov.in/planningcommission.gov.in/docs/plans/planrel/fiveyr/3rd/3planch32.html> |
| 15. | Planning Commission (PC) 4 [17] | 1969 | Health Section of the fourth Five Year Plan (1969-74) | <https://niti.gov.in/planningcommission.gov.in/docs/plans/planrel/fiveyr/4th/4planch18.html> |
| 16. | Planning Commission (PC) 5 [18] | 1974 | Health Section of the fifth Five Year Plan (1974-79) | <https://niti.gov.in/planningcommission.gov.in/docs/plans/planrel/fiveyr/5th/5planch5.html> |
| 17. | Planning Commission (PC) 6 [19] | 1980 | Health Section of the sixth Five Year Plan (1980-85) | <https://niti.gov.in/planningcommission.gov.in/docs/plans/planrel/fiveyr/6th/6planch22.html> |
| 18. | Planning Commission (PC) 7 [20] | 1985 | Health Section of the seventh Five Year Plan (1985-90) | <https://niti.gov.in/planningcommission.gov.in/docs/plans/planrel/fiveyr/7th/vol2/7v2ch11.html> |
| 19. | Planning Commission (PC) 8 [21] | 1992 | Health Section of the eighth Five Year Plan (1992-97) | <https://niti.gov.in/planningcommission.gov.in/docs/plans/planrel/fiveyr/8th/vol2/8v2ch12.htm> |
| 20. | Planning Commission (PC) 9 [22] | 1997 | Health Section of the ninth Five Year Plan (1997-2002) | <https://niti.gov.in/planningcommission.gov.in/docs/plans/planrel/fiveyr/9th/vol2/v2c3-4.htm> |
| 21. | Planning Commission (PC) 10 [23] | 2002 | Health Section of the tenth Five Year Plan (2002-07) | <https://niti.gov.in/planningcommission.gov.in/docs/plans/planrel/fiveyr/10th/volume2/v2_ch2_8.pdf> |
| 22. | Planning Commission (PC) 11 [24] | 2007 | Health Section of the eleventh Five Year Plan (2007-12) | <https://niti.gov.in/planningcommission.gov.in/docs/plans/planrel/fiveyr/11th/11_v2/11v2_ch3.pdf> |
| 23. | Planning Commission (PC) 12 [25] | 2012 | Health Section of the twelfth Five Year Plan (2012-17) | <http://nhm.gov.in/images/pdf/publication/Planning_Commission/12th_Five_year_plan-Vol-3.pdf> |
| 24. | High Level Expert Group (HLEG) Report  [26] | 2011 | Key recommendations of the High Level Expert Group (HLEG), constituted in 2010 by the Planning Commission of India. | <http://nhm.gov.in/images/pdf/publication/Planning_Commission/rep_uhc0812.pdf> |
| 25. | National Rural Health Mission (NRHM) Report  [27] | 2005 | Document outlining the components of the National Rural Health Mission (NRHM) which focuses on the rural population of the country. | <https://nhm.gov.in/WriteReadData/l892s/nrhm-framework-latest.pdf> |
| 26. | National Urban Health Mission (NUHM) Report  [28] | 2013 | Document outlining the National Urban Health Mission (NUHM), a sub-mission of the broader National Health Mission (NHM). | <http://nhm.gov.in/images/pdf/NUHM/Implementation_Framework_NUHM.pdf> |
| 27. | Indian Public Health Standard guidelines (IPHS)- District Hospitals (DH)  [29] | 2012 | Standard set of guidelines for district hospitals. | <http://nhm.gov.in/images/pdf/guidelines/iphs/iphs-revised-guidlines-2012/district-hospital.pdf> |
| 28. | Indian Public Health Standard guidelines (IPHS)- Sub-District Hospitals (SDH)  [30] | 2012 | Standard set of guidelines for sub-district hospitals. | <http://nhm.gov.in/images/pdf/guidelines/iphs/iphs-revised-guidlines-2012/sub-district-sub-divisional-hospital.pdf> |
| 29. | Indian Public Health Standard guidelines (IPHS)- Community Health Centres (CHCs) [31] | 2012 | Standard set of guidelines for community health centres. | <http://nhm.gov.in/images/pdf/guidelines/iphs/iphs-revised-guidlines-2012/community-health-centres.pdf> |
| 30. | Indian Public Health Standard guidelines (IPHS)- Primary Health Centres (PHCs)  [32] | 2012 | Standard set of guidelines for primary health centres. | <http://nhm.gov.in/images/pdf/guidelines/iphs/iphs-revised-guidlines-2012/primay-health-centres.pdf> |
| 31. | Indian Public Health Standard guidelines (IPHS)- Sub-Centres (SCs)  [33] | 2012 | Standard set of guidelines for sub-centres. | <http://nhm.gov.in/images/pdf/guidelines/iphs/iphs-revised-guidlines-2012/sub-centers.pdf> |
| 32. | National Blood Policy (NBP)  [34] | 2007 | Guidelines released by the National Blood Transfusion Council (NBTC) to ensure access to safe and quality blood. | <http://www.naco.gov.in/sites/default/files/National%20Blood%20Policy_0.pdf> |
| 33. | National Programme for Health Care of the Elderly (NPHCE) Report  [35] | 2010 | The report provides a comprehensive summary of the national program of the same name. | <https://dghs.gov.in/content/1359_3_NationalProgrammeHealthCareElderly.aspx> |
| 34. | National Council for Clinical Establishments (NCCE) Report  [36] | 2010 | The act was introduced with the purpose of maintaining minimum standards for clinical establishments. | <https://dghs.gov.in/content/1361_3_NationalCouncilClinicalEstablishments.aspx> |
| 35. | National Oral Health Programme (NOHP) Report  [37] | 2014 | Report of the National Oral Health Programme (NOHP) which attempts to enable access to affordable oral health for all. | <https://dghs.gov.in/content/1352_3_NationalOralHealthProgramme.aspx> |
| 36. | National Programme for Palliative Care (NPPC) Report  [38] | 2012 | Document highlighting the key features of the National Program for Palliative Care (NPPC) which aims to provide care for terminal cases of cancer, AIDS, etc. | <https://dghs.gov.in/content/1351_3_NationalProgramforPalliativeCare.aspx> |
| 37. | National Programme for Prevention and Management of Trauma and Burn Injuries (NPPMBI) Report  [39] | 2013 | Document providing an overview of the National Programme for Prevention and Management of Trauma and Burn Injuries (NPPMBI) which encompasses the creation of trauma care facilities and recruitment of trained personnel. | <https://dghs.gov.in/content/1528_3_NationalProgrammeforPreventionandManagement.aspx> |
| 38. | National Organ Transplant Programme (NOTP) Report  [40] | 2015 | Report outlining the key features of NOTP, with the goal of improving access to life-saving organ transplantation. | <https://dghs.gov.in/content/1353_3_NationalOrganTransplantProgramme.aspx> |
| 39. | National Programme for Control of Blindness and Visual Impairment (NPCBVI) Report  [41] | 1976 | Report for the NPCBVI programme which aims to reduce the prevalence of blindness. | <https://dghs.gov.in/content/1354_3_NationalProgrammeforControlofBlindnessVisual.aspx> |
| 40. | National Programme for Prevention and Control of Cancer, Diabetes, Cardiovascular Diseases and Stroke (NPCDCS) Report  [42] | 2010 | The NPCDCS programme was launched in 2010 and released its report which dealt with the control of non-communicable diseases. | <https://dghs.gov.in/content/1363_3_NationalProgrammePreventionControl.aspx> |

**Supplementary References**

1. Bhore J, Amesur RA, Banerjea AC, Butt AH, Chandrachud RB, Dadabhoy DJR, et al. Report of the Health Survey and Development Committee : Volume 1 [Internet]. Government of India Press; 1946. Available: http://www.nihfw.org/Doc/Reports/bhore%20Committee%20Report%20VOL-1%20.pdf

2. Bhore J, Amesur RA, Banerjea AC, Butt AH, Chandrachud RB, Dadabhoy DJR, et al. Report of the Health Survey and Development Committee : Volume 2 [Internet]. Government of India Press; 1946. Available: http://www.nihfw.org/Doc/Reports/Bhore%20Committee%20Report%20-%20Vol%20II.pdf

3. Bhore J, Amesur RA, Banerjea AC, Butt AH, Chandrachud RB, Dadabhoy DJR, et al. Report of the Health Survey and Development Committee: Volume 3 [Internet]. Government of India Press; 1946. Available: https://www.nhp.gov.in/sites/default/files/pdf/Bhore_Committee_Report-3.pdf

4. Mudaliar AL, Rao T, Melkote GS, Srinivasan V, Chakravarti DN, Rao KN, et al. Report of the Health Survey and Planning Committee [Internet]. Government of India Press; 1962. Available: https://www.nhp.gov.in/sites/default/files/pdf/Mudalier_Vol.pdf

5. Mukerji B. Mukerji Committee Report I [Internet]. Government of India Press; 1965. Available: https://www.nhp.gov.in/sites/default/files/pdf/Mukerjee_Committee_Report.pdf

6. Mukerji B. Mukerji Committee Report II [Internet]. Government of India Press; 1966. Available: https://www.nhp.gov.in/sites/default/files/pdf/Mukerji_Committee_Report_PartII.pdf

7. Jungalwalla N, Bhatia D, Sharma DN, Sharma HM, Mukerjee CL, Patel TB, et al. Report of the Committee on Integration of Health Services [Internet]. Directorate General of Health Services, Government of India; 1967. Available: https://www.nhp.gov.in/sites/default/files/pdf/Jungalwal_Committee_Report.pdf

8. Singh K. Report of the Committee on Multipurpose workers under Health and Family Planning Programme [Internet]. Government of India Press; 1973. Available: https://www.nhp.gov.in/sites/default/files/pdf/Kartar_Singh_Committee_Report.pdf

9. Shrivastav DJ., Gopalan DC, Ramalingaswami V, Chuttani DP., Naik JP, Krishnamurthi CR, et al. Report of the Group on Medical Education and Support Manpower  [Internet]. Government of India Press; 1973. Available: https://www.nhp.gov.in/sites/default/files/pdf/Srivastava_Committee_Report.pdf

10. Bajaj JS. Bajaj Committee Report  [Internet]. Government of India Press; 1986. Available: https://www.nhp.gov.in/sites/default/files/pdf/Bajaj_Committee_report.pdf

11. Ministry of Health and Family Welfare. National Health Policy [Internet]. Government of India; 1983. Available: https://www.nhp.gov.in/sites/default/files/pdf/nhp_1983.pdf

12. Ministry of Health and Family Welfare. National Health Policy [Internet]. Government of India; 2002. Available: https://www.nhp.gov.in/sites/default/files/pdf/NationaL_Health_Pollicy.pdf

13. Ministry of Health and Family Welfare. National Health Policy [Internet]. Government of India; 2017. Available: https://www.nhp.gov.in/nhpfiles/national_health_policy_2017.pdf

14. Nehru J, Nanda G, Krishnamachari VT, Deshmukh C, Mehta GL, Patil RK, et al. First Five Year Plan [Internet]. Government of India Press; 1951. Available: https://niti.gov.in/planningcommission.gov.in/docs/plans/planrel/fiveyr/1st/1pconclu.html

15. Nehru J, Krishnamachari VT, Nanda G, Deshmukh C, Neogy KC, Ghosh JC, et al. Second Five Year Plan  [Internet]. Government of India Press; 1956. Available: https://niti.gov.in/planningcommission.gov.in/docs/plans/planrel/fiveyr/2nd/2planch25.html

16. Nehru J, Nanda G, Desai M, Menon VKK, Trivedi CM, Singh TN, et al. Third Five Year Plan  [Internet]. Government of India Press; 1961. Available: https://niti.gov.in/planningcommission.gov.in/docs/plans/planrel/fiveyr/3rd/3pconclu.html

17. Gandhi I, Gadgil DR, Venkataraman R, Venkatappiah B, Pant P, Chaudhuri BDN, et al. Fourth Five Year Plan [Internet]. Government of India Press; 1969. Available: https://niti.gov.in/planningcommission.gov.in/docs/plans/planrel/fiveyr/4th/4planch18.html

18. Gandhi I. Fifth Five Year Plan [Internet]. Government of India Press; 1974. Available: https://niti.gov.in/planningcommission.gov.in/docs/plans/planrel/fiveyr/5th/5planch5.html

19. Gandhi I, Tiwari ND, Venkataraman R, Swaminathan MS, Fazal M, Singh M. Sixth Five Year Plan [Internet]. Government of India Press; 1980. Available: https://niti.gov.in/planningcommission.gov.in/docs/plans/planrel/fiveyr/6th/6planch22.html

20. Gandhi R, Singh M, Rao PVN, Singh V, Singh B, Rao CHH, et al. Seventh Five Year Plan [Internet]. Government of India Press; 1985. Available: https://niti.gov.in/planningcommission.gov.in/docs/plans/planrel/fiveyr/7th/vol2/7v2ch11.html

21. Rao PVN, Mukherjee P, Singh M, Pawar S, Jakhar B, Bhardwaj HR, et al. Eighth Five Year Plan [Internet]. Government of India Press; 1992. Available: https://niti.gov.in/planningcommission.gov.in/docs/plans/planrel/fiveyr/8th/vol2/8v2ch12.htm

22. Vajpayee AB. Ninth Five Year Plan [Internet]. Government of India Press; 1997. Available: https://niti.gov.in/planningcommission.gov.in/docs/plans/planrel/fiveyr/9th/vol2/v2c3-4.htm

23. Vajpayee AB. Tenth five Year Plan [Internet]. Government of India Press; 2002. Available: https://niti.gov.in/planningcommission.gov.in/docs/plans/planrel/fiveyr/10th/volume2/v2_ch2_8.pdf

24. Singh DM. Eleventh Five Year Plan [Internet]. Government of India Press; 2007. Available: https://niti.gov.in/planningcommission.gov.in/docs/plans/planrel/fiveyr/11th/11_v2/11v2_ch3.pdf

25. Singh DM. Twelfth Five Year Plan [Internet]. Government of India Press; 2017. Available: http://nhm.gov.in/images/pdf/publication/Planning_Commission/12th_Five_year_plan-Vol-3.pdf

26. Thakur J. Key recommendations of high-level expert group report on universal health coverage for India. Indian J Community Med. 2011;36: S84-5.

27. Ministry of Health and Family Welfare. National Rural Health Mission : Framework for Implementation [Internet]. Government of India; 2005. Available: https://nhm.gov.in/WriteReadData/l892s/nrhm-framework-latest.pdf

28. Ministry of Health and Family Welfare. National Urban Health Mission: Framework for Implementation [Internet]. New Delhi: Government of India; 2013 May. Available: http://nhm.gov.in/images/pdf/NUHM/Implementation_Framework_NUHM.pdf

29. Directorate General of Health Services, Ministry of Health and Family Welfare. Indian Public Health Standards (IPHS) Guidelines for District Hospitals Revised 2012 [Internet]. Directorate General of Health Services, Ministry of Health and Family Welfare, Government of India; 2012. Available: http://nhm.gov.in/images/pdf/guidelines/iphs/iphs-revised-guidlines-2012/district-hospital.pdf

30. Directorate General of Health Services, Ministry of Health and Family Welfare. Indian Public Health Standards (IPHS) Guidelines for Sub-District Hospitals Revised 2012 [Internet]. Directorate General of Health Services, Ministry of Health and Family Welfare, Government of India; 2012. Available: http://nhm.gov.in/images/pdf/guidelines/iphs/iphs-revised-guidlines-2012/sub-district-sub-divisional-hospital.pdf

31. Directorate General of Health Services, Ministry of Health and Family Welfare. Indian Public Health Standards (IPHS) Guidelines for Community Health Centres Revised 2012. Directorate General of Health Services, Ministry of Health and Family Welfare, Government of India; 2012.

32. Directorate General of Health Services, Ministry of Health and Family Welfare. Indian Public Health Standards (IPHS) Guidelines for Primary Health Centres Revised 2012 [Internet]. Directorate General of Health Services, Ministry of Health and Family Welfare, Government of India; 2012. Available: http://nhm.gov.in/images/pdf/guidelines/iphs/iphs-revised-guidlines-2012/primay-health-centres.pdf

33. Directorate General of Health Services, Ministry of Health and Family Welfare. Indian Public Health Standards (IPHS) Guidelines for Sub-Centres Revised 2012 [Internet]. Directorate General of Health Services, Ministry of Health and Family Welfare, Government of India; 2012. Available: http://nhm.gov.in/images/pdf/guidelines/iphs/iphs-revised-guidlines-2012/sub-centers.pdf

34. National Aids Control Organization (NACO). National Blood Policy [Internet]. Government of India; 2007. Available: http://www.naco.gov.in/sites/default/files/National%20Blood%20Policy_0.pdf

35. Directorate General of Health Services, Ministry of Health and Family Welfare. National Programme for Health Care of the Elderly [Internet]. Directorate General of Health Services, Ministry of Health and Family Welfare, Government of India; 2010. Available: https://dghs.gov.in/content/1359_3_NationalProgrammeHealthCareElderly.aspx

36. Directorate General of Health Services, Ministry of Health and Family Welfare. National Council for Clinical Establishments [Internet]. Directorate General of Health Services, Ministry of Health and Family Welfare, Government of India; 2010. Available: https://dghs.gov.in/content/1361_3_NationalCouncilClinicalEstablishments.aspx

37. Directorate General of Health Services, Ministry of Health and Family Welfare. National Oral Health Programme [Internet]. Directorate General of Health Services, Ministry of Health and Family Welfare, Government of India; 2014. Available: https://dghs.gov.in/content/1352_3_NationalOralHealthProgramme.aspx

38. Directorate General of Health Services, Ministry of Health and Family Welfare. National Program for Palliative Care  [Internet]. Directorate General of Health Services, Ministry of Health and Family Welfare, Government of India; 2012. Available: https://dghs.gov.in/content/1351_3_NationalProgramforPalliativeCare.aspx

39. Directorate General of Health Services, Ministry of Health and Family Welfare. National Programme for Prevention and Management of Trauma and Burn Injuries [Internet]. Directorate General of Health Services, Ministry of Health and Family Welfare, Government of India; 2013. Available: https://dghs.gov.in/content/1528_3_NationalProgrammeforPreventionandManagement.aspx

40. Directorate General of Health Services, Ministry of Health and Family Welfare. National Organ Transplant Programme [Internet]. Directorate General of Health Services, Ministry of Health and Family Welfare, Government of India; 2015. Available: https://dghs.gov.in/content/1353_3_NationalOrganTransplantProgramme.aspx

41. Directorate General of Health Services, Ministry of Health and Family Welfare. National Programme for Control of Blindness & Visual Impairment [Internet]. Directorate General of Health Services, Ministry of Health and Family Welfare, Government of India; 1976. Available: https://dghs.gov.in/content/1354_3_NationalProgrammeforControlofBlindnessVisual.aspx

42. Directorate General of Health Services, Ministry of Health and Family Welfare. National Programme for Prevention and Control of Cancer, Diabetes,Cardiovascular Diseases and Stroke [Internet]. Directorate General of Health Services, Ministry of Health and Family Welfare, Government of India; 2010. Available: https://dghs.gov.in/content/1363_3_NationalProgrammePreventionControl.aspx
