# Supplementary material for: Prioritization of surgical, obstetric, trauma, and anesthesia care in India over seven decades: A systematic analysis of policy documents
Source: PLOS Glob Public Health. 2023 Jul 31;3(7):e0002084. doi: 10.1371/journal.pgph.0002084 (PMC10389714; doi:10.1371/journal.pgph.0002084)
Supplement: Attachment — Submitted filename: 28_JUN_23_SZ_response_letter_surgical_policy_plosgph.docx [file pgph.0002084.s003.docx]

Response Letter

Journal Requirements:

1. Please provide separate figure files in .tif or .eps format.

For more information about figure files please see our guidelines: LINK

https://journals.plos.org/globalpublichealth/s/figures

https://journals.plos.org/globalpublichealth/s/figures#loc-file-requirements

**Authors’ response: The revised submission includes all figure files in .tif format.**

2. We have noticed that you have uploaded Supporting Information files, but you have not included a list of legends. Please add a full list of legends for your Supporting Information files after the references list.

**Authors’ response: These have been added in the revised manuscript on page 23.**

Please review your reference list to ensure that it is complete and correct. If you have cited papers that have been retracted, please include the rationale for doing so in the manuscript text, or remove these references and replace them with relevant current references. Any changes to the reference list should be mentioned in the rebuttal letter that accompanies your revised manuscript. If you need to cite a retracted article, indicate the article’s retracted status in the References list and also include a citation and full reference for the retraction notice.

**Authors’ response: We have checked all the references and the bibliography. We do not believe that there are any references to retracted articles.**

Reviewers’ comments:

Reviewer #1: Very interesting article highlighting the significant need to include SOTA priorities in policy documents to enact change at a national level.

**Authors’ response: We thank the reviewer for this comment and we are glad that they found our work interesting.**

Would be interesting to see if adding additional surgical terms or adding weight to more important terms changes the findings.

**Authors’ response: We agree with the reviewer. However, the current manuscript uses keywords or terms that have been previously used and tested across multiple such analyses in other settings. An important reason to choose the keyword list from previous studies was to be able to have these findings comparable. Coming up with new keywords will require feedback from and consensus among multiple stakeholders, which might be beyond the scope of this study.**

**We did not use differential weighting in this study as deciding over weights would require inputs from experts. Further, arbitrary weights can skew findings or reduce their interpretability. Hence, we considered non-controversial equal weights for calculating normalized metrics. However, we present analyses across different subgroups (see Figure 8) and also present keyword-wise mentions (see Table 3) for the readers to see the importance of specific issues and keywords.**

**In the revised manuscript, we have added the points raised by the reviewer in Section 4 - Discussion, Sub-section 4.4 - Strengths, Limitations, and Future Directions on page 17.**

Reviewer #2: This study aims to assess the level of prioritization afforded to surgical, obstetric, trauma and anesthesia care in health policy, planning and programmatic documents in India. Overall, the study is a useful contribution to the literature on global surgery policy, particularly in Asia where global surgery policy research has been lacking. The aims for the study are clear and the methodology used in appropriate, building on previous methodology used in other studies in Africa. It is also a more comprehensive analysis that previous studies. I commend the authors for this.

**Authors’ response: We thank the reviewer for this comment and we are glad that they found our work interesting.**

Minor edits/suggestions

- I understand that Hindi and English are the official languages of India. In focusing only on policies in English language, is it possible that some policies may have been excluded from the analysis?

**Authors’ response: We thank the reviewer for this comment. We included documents in published English for easy, consistent, and reproducible screening. However, the Government of India usually releases policy documents in both languages (English and Hindi/regional languages) simultaneously. For reference, see the English (**[**https://main.mohfw.gov.in/sites/default/files/9147562941489753121.pdf**](https://main.mohfw.gov.in/sites/default/files/9147562941489753121.pdf)**) and Hindi (**[**https://main.mohfw.gov.in/sites/default/files/80739461831490962679.pdf**](https://main.mohfw.gov.in/sites/default/files/80739461831490962679.pdf)**) versions of the National Health Policy (2017) are archived in the same portal. At times, the content in different languages is presented in the same document. Hence, while an understandable concern, it is unlikely that any documents were missed out in this case.**

**Regardless, we have added this as a limitation in the revised manuscript. See Section 4 - Discussion, Sub-section 4.4 - Strengths, Limitations, and Future Directions on page 17.**

- Did the authors consider reaching out to the Ministry of Health for additional policy documents that they might have missed by searching online? It is often the case that not all policy documents are available online.

**Authors’ response: We thank the reviewer for this comment. We did not reach out to the Ministry for acquiring any additional documents. In the current analysis, we wanted to include the official and completely drafted final version of documents released by the government that are freely available in the public domain. In part, this ensured that our analysis is reproducible and transparent. Additionally, the ministries of the Government of India release all policy-related material on their official websites and/or digital archives as it is required by law.**

**Regardless, we have added this as a limitation in the revised manuscript. See Section 4 - Discussion, Sub-section 4.4 - Strengths, Limitations, and Future Directions on page 17.**

- Overall the discussions and limitations mentioned are comprehensive and useful. However, it might be helpful if the authors could hypothesize why SOTA prioritization in health policy document is low in India despite the high burden of conditions requiring surgical care. They do mention the little focus on information management but perhaps there is more to this. The discussion section could be strengthened even further if the authors would consider expanding on why prioritization is low.

**Authors’ response: We thank the reviewer for this comment. We have included a paragraph on potential reasons for low SOTA care prioritization in India under Section 4 - Discussion, Sub-section 4.3 - Interpretation and relevance of findings on page 16.**

Have there been any SOTA advocacy efforts in the past or currently which may explain the results?

**Authors’ response: We thank the reviewer for this comment. Our results depict overall low prioritization. As noted in the manuscript, this is in part due to limited advocacy in the past. However, there have been some recent efforts that have been previously discussed by us elsewhere (https://gh.bmj.com/content/7/11/e010292). We have cited the relevant literature in Section 4 - Discussion, Sub-section 4.3 - Interpretation and relevance of findings on page 16.**

Perhaps the authors could consider using a framework like the Shiffman and Smith frame work on political priority for global health initiatives ([https://www.thelancet.com/journals/lancet/article/PIIS0140-6736(07)61579-7/fulltext](https://urldefense.com/v3/__https://www.thelancet.com/journals/lancet/article/PIIS0140-6736(07)61579-7/fulltext__;!!OToaGQ!osKPgTqhw-riJRqLHmMk_KWJjpC-xanBTGpWooFQ3RJXw7A72_E-_7ScVC_3KHWdtMTb-QFW96RqyK9Zk2ffCA$)) or other similar frameworks to enhance the discussion of key findings.

**Authors’ response: We thank the reviewer for this comment. Using a framework like the Shiffman and Smith framework would require acquiring information about the policy processes including development and implementation directly from policy makers. While important, it be would beyond the scope of our current document analysis. We agree that there is a need for such an assessment. Hence, we have included it as a suggestion under Section 4 - Discussion, Sub-section 4.3 - Interpretation and relevance of findings on page 16.**

- I commend the authors on a well articulated and comprehensive limititation section

**Authors’ response: We thank the reviewer for this comment.**
